# The effects of acute exercise intensity on memory: Controlling for state-dependence

**DOI:** 10.3758/s13421-024-01660-2

**Published:** 2024-11-14

**Authors:** Paul Loprinzi, Lauren Fuglaar, Rylie Mangold, Sierra Petty, Myungjin Jung, L. B. Day, Zakary Patrick, Kirk I. Erickson, William L. Kelemen

**Affiliations:** 1 Department of Health, Exercise Science and Recreation Management, University of Mississippi, Oxford, MS 38655 USA; 2 Department of Psychology, University of Mississippi, Oxford, MS USA; 3 Department of Biology, University of Mississippi, Oxford, MS USA; 4 Department of Kinesiology, Health Promotion and Recreation, University of North Texas, Denton, TX USA; 5 Department of Biology and Interdisciplinary Neuroscience Minor, University of Mississippi, Oxford, MS USA; 6 Neuroscience Institute, AdventHealth Research Institute, Orlando, FL USA; 7 Department of Psychology, University of Pittsburgh, Pittsburgh, PA USA; 8 Department of Psychology, Texas State University, San Marcos, TX USA

**Keywords:** Dual task, Cognition, Episodic memory, Exercise

## Abstract

The present experiment evaluated the effects of varying intensities of acute exercise on free-recall memory performance while controlling for potential state-dependent effects. Forty-eight young adults completed a within-subject experiment involving seven primary laboratory visits. The encoding and retrieval phases were matched or mismatched by taking place either during rest or during a less than 5-min bout of acute exercise, and at moderate or vigorous intensity. We did not find evidence that the effects of acute exercise on memory were state-dependent but instead demonstrated that memory recall was greater when memory retrieval occurred during vigorous-intensity exercise compared to rest. These findings have important implications for the strategic placement of exercise during the phases of memory (e.g., acquisition, storage, retrieval) to optimize memory performance and suggest boundary conditions of state-dependent learning. We discuss various theoretical accounts (e.g., shift in metabolic resources across brain regions) to explain these findings.

## Introduction

Acute exercise can either improve or impair memory performance (Cantelon & Giles, 2021); such effects have been observed with a variety of exercise modalities (e.g., treadmill, cycling) and memory assessments (e.g., free recall, recognition) (Loprinzi et al., 2019a). These effects vary based on several factors, such as when the acute bout of exercise occurs in relation to the different phases of memory, for example, exercise occurring before encoding, during encoding, during consolidation, during memory retrieval, or occurring at multiple times during these phases (e.g., during both encoding and retrieval to control for state dependence) (Loprinzi, Day et al., 2021a, Loprinzi, Roig et al., 2021b). Other critical factors that influence whether acute exercise improves or impairs memory is the intensity and duration of acute exercise, and these additional factors may interact with the memory phase in which the exercise bout occurs (Loprinzi et al., 2019a). The present experiment attempts to disentangle some of these issues to improve our understanding of when we expect acute exercise to influence memory.

### Potential mechanisms of acute exercise on memory

Although additional mechanistic work is needed, especially in humans, it is thought that exercise prior to encoding may improve encoding and consolidation processes (El-Sayes et al., 2019). As discussed in detail elsewhere (Loprinzi, Roig et al., 2021b), acute exercise may improve encoding processes by facilitating the allocation of attentional resources during learning. This benefit might be partly attributed to an increase in cerebral blood flow for the delivery of essential oxygen and nutrients crucial for optimal brain function (Mulser & Moreau, 2023). Acute exercise – prior to encoding or during early consolidation – may improve consolidation processes by, for example, upregulating key neurotransmitters (e.g., noradrenaline) and neurotrophins (e.g., brain-derived neurotrophic factor) to help stabilize the memory trace (for reviews, see Loprinzi et al., 2017, 2018). Further, unlike chronic stress, exercise-induced increases in glucocorticoids to meet metabolic demands may improve cognition via their effects on increasing dopamine in the medial prefrontal cortex regardless of whether exercise is acute or chronic (Chen et al., 2017). Additionally, exercise promotes neurogenesis in the rodent hippocampus and, in humans, modifies connectivity between pivotal brain regions, particularly between the hippocampus and the prefrontal cortex, which are intrinsically linked with memory consolidation (Voss et al., 2019, 2020).

In addition to these psychological (e.g., enhanced attention during encoding and/or retrieval) and neurophysiological (e.g., increased production of key growth factors and neurotransmitters) mechanisms, certain types of motor functions may influence memory if specific sensorimotor conditions are congruent or incongruent during encoding and retrieval (Iani, 2019). Per the sensorimotor model, during encoding, we register perceptual and motor information, and successful retrieval of this information involves the reactivation of these representations (Iani, 2019). Similar to state-dependent theories of memory (Tulving & Thomson, 1973), reinstating the body posture (e.g., lying down in a recliner) or movement pattern that occurred during encoding of the event (e.g., memory at a dentist) has been shown to enhance autobiographical memory performance (Dijkstra et al., 2007).

To better understand the effects of exercise on memory from sensorimotor and state-dependent accounts, it is important to understand the brain regions activated during exercise, auditory encoding, and verbal retrieval of words, all design features of the present experiment. Although minimal work has evaluated brain regions activated during treadmill running (Nordin et al., 2019), studies have evaluated brain regions activated during cycling exercise in humans. For example, Fontes et al. (2015) showed that when cycling at a self-perceived high intensity, the posterior cingulate gyrus and precuneus become activated. In their follow-up study, Fontes et al. (2020) demonstrated that, during incremental cycling exercise, various brain regions were activated and deactivated during self-perceived low and high exercise intensities. As an example, at a low intensity, the cerebellum was activated, whereas the dorsolateral prefrontal cortex was deactivated. At a high intensity, the pre- and post-central gyrus and medial frontal gyrus were activated, whereas the dorsolateral prefrontal cortex, middle temporal gyrus, and parahippocampal gyrus were deactivated. In contrast, other work demonstrates that, during high-intensity cycling (75% of VO_2_ max), brain glucose levels decrease in the cerebellum, superior and middle frontal cortex, temporal cortex, thalamus, and anterior cingulate (Kemppainen et al., 2005).

Brain regions involved in encoding and retrieving studied items include various neural structures that may be activated from acute exercise. For example, during encoding, the left prefrontal cortex, left/bilateral medial temporal lobe, left temporal auditory cortex and right cerebellum are activated; brain regions involved for item recall include the left prefrontal motor regions for speech, right prefrontal cortex, bilateral motor areas, and cerebellum (Cabeza & Nyberg, 2000). These findings suggest that acute exercise may have a unique effect on memory by activating specific brain regions during exercise, some of which also appear to be activated during encoding and retrieval. However, these assertions need to be cautiously considered given the variability across studies regarding region-specific brain activation/deactivation from exercise, differing methodologies regarding exercise intensity in these studies, and incomplete congruence in region-specific brain activation during exercise and during encoding/retrieval.

Collectively, the underlying mechanisms through which acute exercise may influence memory are complex and likely occurring across multiple levels and systems. Higher-order brain structures, such as the prefrontal cortex and hippocampus, are strong candidates for structures that regulate the impact of exercise on memory (Loprinzi et al., 2017; Moore & Loprinzi, 2021), but more recently, the cerebellum has been shown to play a critical role in sensorimotor and cognitive encoding and memory processes, and integration of procedural processes at motor, sensory, and cognitive levels (Guell et al., 2018; Iani, 2019).

### The timing of acute exercise on memory

Accumulating research demonstrates beneficial effects of acute exercise on free-recall episodic memory function (Coles & Tomporowski, 2008; Labban & Etnier, 2011, 2018; Roig et al., 2013; Slutsky-Ganesh et al., 2020), defined as the retrospective recall of past events or episodes. The timing of the bout of exercise, however, plays an important role (Roig et al., 2016). When exercise occurs shortly before memory encoding – or even during the early stages of consolidation – memory benefits may ensue (Loprinzi, Day et al., 2021a, Loprinzi, Roig et al., 2021b). This memory benefit has been observed for both moderate- and vigorous-intensity treadmill exercise (Loprinzi et al., 2022a, 2022b), and typically occurs in studies implementing an exercise duration of 20–30 min (Loprinzi et al., 2019a). Less research, however, has evaluated the effects of even shorter bouts (e.g., < 10 min) of acute exercise on memory (Suwabe et al., 2018), which is worth considering given the practical utility of integrating very brief periods of movement throughout the day. Memory benefits may even occur within 15-min post-treadmill exercise (Loprinzi et al., 2022a, 2022b) and have long-lasting effects beyond a day (Loprinzi, Day et al., 2021a, Loprinzi, Roig et al., 2021b).

Placing the bout of acute exercise during the early consolidation window or right before retrieval has also been shown to enhance long-term free-recall memory performance (Loprinzi, Day et al., 2021a, Loprinzi, Roig et al., 2021b). In contrast, when the bout of exercise occurs *during* the encoding process, cognition (including memory) may be impaired, especially if this dual-task situation (i.e., completing two tasks simultaneously) occurs during vigorous-intensity exercise (Jung et al., 2022; Tomporowski & Qazi, 2020). In alignment with the transient hypofrontality model (Dietrich, 2006; Dietrich & Audiffren, 2011), this dual-task scenario may re-allocate attentional and metabolic resources away from encoding the to-be-remembered information and direct such resources toward brain structures (e.g., motor cortex, basal ganglia, and cerebellum) involved in motor planning, voluntary movement, and timing and sequencing of movement, respectively. However, prior work suggests that simultaneous demands that attract resources away from the memory task during encoding will impair encoding processes, but dual-task effects may have less of a detrimental effect on retrieval-based processes (Logie et al., 2007). Such dual-task interference during retrieval may be more likely to occur if there is an overlap in the processes involved in memory retrieval and the secondary task – in this case, exercise. Although it is conceivable that exercise (running specifically) could prime processes involved in memory retrieval, performing a well-developed exercise skill, such as running, is unlikely to require memory retrieval. Thus, given minimal overlap in processes regulating memory retrieval and running, dual-task interference effects are less likely to occur when memory retrieval occurs during exercise, especially if the memory retrieval task is unrelated to motor processes or imagined sensorimotor actions.

### State dependency

State-dependent learning involves a match in emotional or physiological state or physical context between encoding and retrieval. We implemented a state-dependent design in which matched (e.g., rest during both encoding and retrieval or exercise during both encoding and retrieval) and mismatched (e.g., rest during encoding with exercise during retrieval or vice-versa) conditions were evaluated. Notably, such state-dependent effects have been extensively investigated in the field of experimental psychology (Kelemen & Creeley, 2003; Tulving & Thomson, 1973). However, these effects have rarely been explored for exercise states (Miles & Hardman, 1998; Salas et al., 2011; Yanes et al., 2019).

### Does memory improve when exercise occurs during encoding and/or retrieval?

The present experiment was designed to evaluate these gaps in the literature by evaluating whether short-duration, acute exercise either improves or impairs free-recall memory when it occurs during encoding and/or during retrieval. We implemented protocols involving both moderate- and vigorous-intensity and included a short exercise duration (less than 5 min) – an exercise duration that has previously been shown to improve cognitive performance (Jaffery et al., 2018).

### Predictions

We anticipate a main effect for encoding, a main effect for retrieval, and an interaction between encoding and retrieval, but anticipate these effects to be observed only for moderate-intensity exercise (replicating Yanes et al., 2019). Thus, we expect the moderate-intensity exercise condition to demonstrate a greater memory benefit when occurring during encoding (main effect for encoding), retrieval (main effect for retrieval), and when there is a match of moderate-intensity exercise occurring during both encoding and retrieval (encoding × retrieval interaction). We do not anticipate any main or interaction effects for vigorous-intensity exercise; vigorous-intensity exercise has been shown to improve memory (Loprinzi et al., 2022a, 2022b), but this memory benefit from vigorous-intensity exercise has been shown to occur when such exercise occurs before – not during – encoding/retrieval.

The present study will provide useful data to contribute theoretical understandings of how acute exercise may impact memory performance. This information will shed light on whether acute exercise elicits state-dependent memory, the extent to which the predictions of the transient hypofrontality (i.e., reduced cognition during vigorous-intensity exercise) extend to durations of about 5 min of vigorous-intensity acute exercise, and provide insights for future experimentation to determine the potential interconnected processes and brain structures (e.g., cerebellum) involved in cognitive and motor function in dual-task situations (e.g., when exercising during encoding/retrieval).

## Methods

### Participants

Forty-eight University of Mississippi students (25 men, 23 women; 18–25 years old) were recruited and took part in this within-subject experimental study; no participants dropped out or had missing data. We recruited as many participants as feasibly possible over a 1-year period of data collection and then computed a sensitivity power analysis (see the *Statistical analyses* section) to determine, based on the sample size collected, what effect size we were powered to detect.

Participants were recruited via word-of-mouth and classroom announcement at the University of Mississippi. Participants were recruited across a variety of majors to increase the generalizability of the participant sample. No compensation was provided for their voluntary participation. Participants were screened to ensure that none of the following conditions were met: (1) self-reported as a daily smoker; (2) self-reported being pregnant; (3) exercised within 5 h of testing; (4) consumed caffeine within 6 h of testing; (5) took medications used to regulate emotion (e.g., SSRIs (selective serotonin reuptake inhibitors)); (6) had a concussion or head trauma within the past 12 months; (7) took marijuana or other mind-altering drugs within the past 2 days; (8) were considered a daily alcohol user (> 30 drinks/month for women; > 60 drinks/month for men) or consumed alcohol in the past 12 h; or (9) answered “yes” to any of the questions on the PAR-Q (Physical Activity Readiness Questionnaire). These criteria were employed to avoid potential confounding effects on memory performance and contraindications to exercise.

### Study design and procedures

Participants completed eight laboratory visits, including an initial visit and then seven main experimental sessions. The first visit included a maximal exercise (treadmill) test (details provided below) and a familiarization to the memory protocol. The heart rate maximum achieved during the first visit was used to set the exercise intensity for the subsequent visits.

After the initial visit, seven subsequent visits occurred. Each participant completed these seven visits in a random order (using a computer-generated algorithm). Allocation concealment occurred by both the researcher and participant not knowing which condition the participant would complete until arriving in the lab. Participants completed Rest-Rest (RR), Rest-Exercise (RE), Exercise-Rest (ER), and Exercise-Exercise (EE) conditions; the first letter denotes what occurred during encoding, while the second letter denotes what took place during recall. For example, RE represents encoding while resting with recall occurring during exercise. The three exercise conditions (RE, ER, and EE) were completed twice, with three involving moderate-intensity exercise and the other three including vigorous-intensity exercise. Further details are noted below. All seven conditions occurred on separate days, at least 24 h apart, and at approximately the same time of day (± 2 h); between-subject assessments varied based on time of day, but within-subject assessments were held relatively constant. Each condition was modeled after other related experiments (Miles & Hardman, 1998; Schramke & Bauer, 1997; Yanes et al., 2019).

**Fig. 1:**
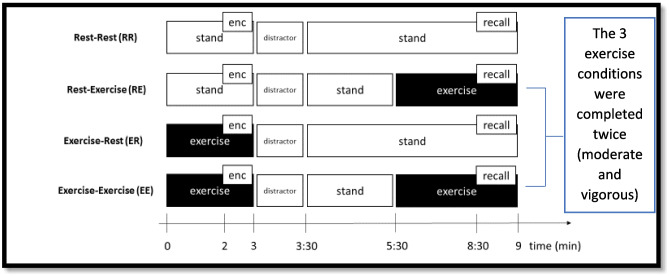
Schematic of the study procedures. Study procedures and involved participants completed eight visits. The first visit (not depicted in this figure) involved a maximal bout of exercise to determine the participant’s maximal heart rate to be used to set the exercise intensity for the subsequent visits. Following this first visit, participants completed seven additional visits, during which subjects encoded a word list (enc), were subjected to a distractor, and then recalled the word list under rest or exercise conditions at times illustrated on the timeline. Subjects participated in one resting visit (RR) and six exercise visits and three exercise conditions (RE, ER, and EE), which were each completed once at moderate-intensity and, on a separate day, at vigorous-intensity

The exact details of these seven main conditions include (see Fig. 1):

- RR: Standing on the treadmill for 3 min with the learning phase starting after 2 min of rest (i.e., they start learning the words at minute 2 of 3); 30-s distractor task; stand (undistracted) for 5 min; and then free recall.
- RE: Standing on the treadmill for 3 min with the learning phase starting after 2 min of rest (i.e., they start learning the words at minute 2 of 3); 30-s distractor task; stand (undistracted) for 2 min; exercise for 3 min; and then memory recall while still exercising.
- ER: Treadmill exercise (walking or running) for 3-min with the learning phase starting after 2 min of exercise (i.e., they start learning the words at minute 2 of 3); after the learning phase, stand and complete 30-s distractor task; stand (undistracted) for 5 min; and then free recall.
- EE: Treadmill exercise (walking or running) for 3 min with the learning phase starting after 2 min of exercise (i.e., they start learning the words at minute 2 of 3); after the learning phase, stand and complete 30-s distractor task; stand (undistracted) for 2 min; exercise for 3 min; and then memory recall while still exercising.

The approximate 30-s distractor task involved the researcher verbally saying five random, non-repeated single digits (0–9), with the participant then asked to recall them back in the order in which they were presented. These distractor data were not collected and therefore are not reported herein as the purpose of this distractor task was to prevent the participant from rehearsing the words immediately after encoding.

### Maximal exercise visit (first visit)

The first laboratory visit included a maximal treadmill-based assessment. The specific assessment included an individualized protocol (Loprinzi et al., 2022a, 2022b). Participants warmed-up for 3 min by walking at 3.5 miles/h (mph). Following this, they engaged in a constant speed throughout the test while the grade increased by 2% every 2 min. After the warm-up period, the speed was set, and remained, at 5.5 mph for the entire exercise protocol. However, if the participant reached the maximum incline of 10%, then the speed was increased by 1 mph every 2 min. The maximal treadmill exercise bout ended when the participant reached exhaustion and indicated they could no longer continue exercising.

### Submaximal exercise sessions

Based on the participant’s maximal heart rate achieved during their maximal exercise bout (visit 1), they exercised at 50% and 80% of their heart rate reserve, respectively, for moderate-intensity and vigorous-intensity exercise. Heart rate reserve was calculated as ([(HR_max_ – HR_rest_) * % target intensity] + HR_rest_). The resting heart rate (before exercise) measurement occurred after sitting quietly for 5 min. During the exercise bout (but not during the rest period between encoding and retrieval), heart rate was measured using a Polar H10 monitor. Other measures of exercise intensity, such as ratings of perceived exertion or volume of oxygen consumption, were not evaluated.

Data collection commenced in January of 2022. Due to concerns with COVID-19, several participants wore a facemask during the entire experimental session. However, this only occurred for the first 2 weeks of data collection, and after this 2-week period, masks were no longer required in the laboratory at the University. Thirteen participants were impacted by this change in protocol. Of these participants, the number of visits during which a mask was worn ranged from one to six (out of eight possible visits). It is unlikely that this change had any negative effect on the data. For example, even during or after walking exercise, wearing a mask does not appear to influence mood or cognitive performance (Caretti, 1999). Similar findings have been shown for light-intensity cycling (Morris et al., 2020). Further, even during vigorous-intensity exercise, wearing a facemask does not appear to induce meaningful effects on the work of breathing, blood gasses, and other physiological parameters (Hopkins et al., 2020).

### Stimulus materials and memory assessment

#### Study phase

Similar to Yanes et al. (2019), participants listened (in a female voice) to (via headphones) a recording of 15 unrelated words, with the words delivered every 1.5 s. The list was played twice, with a 10-s pause in between. Pilot testing ensured that all words could be heard over the sound of the treadmill.

Seven stimulus lists were prepared, with a distinct list (randomly matched and paired for encoding-recall) used for each of the seven conditions, occurring in a random order. The seven lists were generated from the English Lexicon Project using the following constraints, number of syllables (1–3), word length (4–8 letters), frequency (HAL; 50,000-300,000), concreteness (2–5), emotional valence (3–6), and emotional arousal (3–6). These constraints generated 176 words, of which we randomly selected 105 words for the creation of seven lists of 15 words each. These seven lists were not different with regard to number of syllables, *F*(6, 104) = .58, *p* = .75, mean (SE) = 1.50 (.06), word length, *F*(6, 104) = .62, *p* = .72, mean (SE) = 5.22 (.11), word frequency, *F*(6, 104) = 1.58, *p* = .16, mean (SE) = 105,791 (5795.9), concreteness, *F*(6, 104) = .98, *p* = .45, mean (SE) = 3.48 (.07), emotional valence, *F*(6, 104) = .61, *p* = .72, mean (SE) = 5.26 (.06) or emotional arousal, *F*(6, 104) = .77, *p* = .59, mean (SE) = 3.87 (.05). Further, there were very few action or manipulable words, reducing any likelihood that the material in the word list would be encoded with an embodied bias, engaging cerebellar networks that might interfere with any cerebellar dependency in treadmill running during encoding or recall (Iani, 2019).

#### Recall phase

Memory retrieval involved the use of a free recall procedure, as free recall may be more sensitive to exercise (Loprinzi & Caplan, 2024; Moutoussamy et al., 2022) and is more effective in observing a state-dependent learning effect (Eich, 1985; Smith et al., 1978), as opposed to cued-recall or a recognition task. Participants were asked to verbally recall as many words as possible. After 15 s of silence, they were asked to try to recall at least one more word. Two outcomes were assessed, including the number of correct and incorrect words from the condition’s list. Number of correct words was the primary outcome, but to also evaluate whether any observed memory differences across conditions is due to guessing, number of incorrect words was also evaluated. A response was scored as correct if the participant verbally stated the word correctly without making any errors in their response. An incorrect word was defined as any word recalled that was not on the study list for the present condition; intrusion errors from other conditions were not evaluated.

### Statistical analyses

All statistical analyses were computed in JASP (v 18.3). Two-factor repeated-measures ANOVAs (rmANOVAs) were computed, with encoding context and retrieval context serving as the within-subject factors. When the Mauchly’s test of sphericity was statistically significant (*p* < .05), indicating violations to sphericity, the degrees of freedom were corrected with the Huynh-Feldt procedure. Statistical significance was set at *p* < .05. Eta-squared (η^2^) was calculated as an effect size for the ANOVA models.

Specifically, a 2 (encoding: rest vs. exercise) × 2 (retrieval: rest vs. exercise) rmANOVA was computed,^1^ one including moderate-intensity exercise and then a separate 2 × 2 rmANOVA including vigorous-intensity exercise; note, the RR condition was used in both analyses that evaluated exercise intensity separately. We did not include exercise intensity as a factor in a single model because this would have changed the design to a 2 (exercise intensity: moderate v vigorous) × 2 (encoding: rest vs. exercise) × 2 (retrieval: rest vs. exercise) design, ultimately resulting in eight levels (2 × 2 × 2). Our experiment only included seven main conditions (not eight), and to have used this three-factor design in a single model, we would have needed participants to complete an additional rest-rest condition. Since only a single rest-rest condition was implemented in our experiment, this necessitated the use of two separate 2 × 2 designs, one for each exercise intensity. Null frequentist results are supplemented with Bayesian analyses (Bayes factor) to provide evidence of the null model compared to the alternative model. For the Bayesian analyses, the inclusion BF is reported, with BFs between 1 and 3 being anecdotal and > 3 indicating moderate evidence in favor of the alternative (vs. null) hypothesis, whereas BFs between 1 and .33 being anecdotal and < .33 indicating moderate evidence in favor of the null (vs. alternative) hypothesis (Jarosz & Wiley, 2014; Kass & Raftery, 1995). For all Bayesian analyses, the default prior in JASP was used.

The outcomes for these ANOVAs included the number of correct words recalled; separate secondary analyses included the number of incorrect recalled words as the outcome.

Based on a sensitivity analysis, with inputs of an α of 0.05, power of 0.80, 48 participants, seven measurements/conditions, and an assumed repeated-measures correlation of 0.50, there was sufficient power to detect a relatively small effect (i.e., effect size f of 0.14; notably, a small- to medium-effect, respectively, ranges from 0.10 to 0.25).

## Results

### Participants

Demographic and behavioral characteristics of the sample are shown in Table 1. Participants, on average, were 20.5 years of age, roughly equally distributed across biological sex (52% male), within a normal body mass index (24.4 kg/m^2^) and were physically active (255 min/week of self-reported moderate-to-vigorous physical activity).^2^

**Table 1:** Demographic and behavioral characteristics of the participants

Variable	Point estimate	SD
Age, mean years	20.5	1.1
Sex, % men	52.1	
Measured body mass index, mean kg/m^2^	24.4	3.0
Physical activity, mean min/week of MVPA	255.4	188.5
Duration lasted on treadmill for max visit, mean sec	661.0	175.9

*N* = 48

MVPA = moderate-to-vigorous physical activity; SD = standard deviation

### Heart rate responses

The heart rate responses during the experimental visits are shown in Table 2. From the initial visit (max treadmill visit), the mean (SD) maximal heart rate was 191.6 (10.8) beats per minute. Based on this, the calculated target heart rates from the individualized protocol for the moderate-intensity and vigorous-intensity sessions were 136.8 and 169.8 beats per minute, respectively. Heart rates increased when going from rest at encoding to exercise at recall and were also higher during vigorous- compared to moderate-intensity exercise (see Table 2).

**Table 2:** Heart rate (mean/SD) responses (beats per minute) across the experimental sessions

Variable	HR at end of encoding	HR at end of recall	*p*
RR	86.7 (10.0)	86.9 (9.9)	.76
RE Moderate	88.0 (10.0)	134.7 (10.3)	<.001
ER Moderate	135.0 (9.0)	91.4 (11.4)	<.001
EE Moderate	135.4 (8.4)	135.2 (9.6)	.70
RE Vigorous	87.9 (11.1)	165.8 (9.6)	<.001
ER Vigorous	165.1 (10.5)	94.6 (12.1)	<.001
EE Vigorous	165.9 (9.0)	167.9 (8.8)	.002

RR = rest-rest; RE = rest-exercise; ER = exercise-rest; EE = exercise-exercise. Moderate represents the moderate-intensity condition and Vigorous represents the vigorous-intensity condition. P-values were generated from a paired t-test comparing heart rate at the end of encoding to heart rate at the end of recall for each of the seven conditions. Values in parentheses are standard deviations

### Memory performance – correctly recalled words

**Fig. 2:**
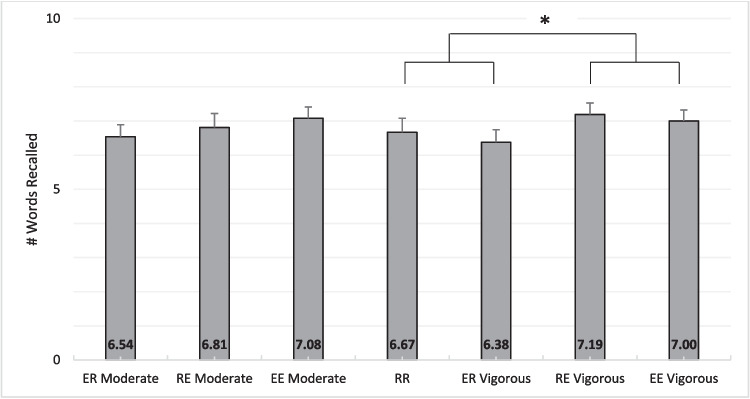
Number of correctly recalled words across conditions. RR = rest-rest; RE = rest-exercise; ER = exercise-rest; EE = exercise-exercise. Moderate represents the moderate-intensity condition and Vigorous represents the vigorous-intensity condition. Error bars are standard errors of the mean. Results showed that there was a main effect for retrieval for vigorous-intensity exercise. In a paired t-test, mean recall was higher for the collapsed RE vigorous and EE vigorous conditions when compared to the collapsed RR and ER vigorous conditions

The number of correct words recalled across the experimental sessions is displayed in Fig. 2. The Mauchly’s test of sphericity for all ANOVA models indicated no violations of sphericity (all *p*s > .05). We found that the number of words recalled did not differ as a function of state-dependent learning for moderate-intensity exercise (Encoding, *F*(1, 47) = .07, *p* = .79, η^2^ < .01, Bayes Factor = .202, Retrieval, *F*(1, 47) = 2.70, *p* = .11, η^2^ = .01, Bayes Factor = .389, interaction *F*(1, 47) = .54, *p* = .47, η^2^ = .004, Bayes Factor = .303). We found that the number of correct words recalled did not differ as a function of state-dependent learning^3^^,^^4^ for vigorous-intensity exercise, but memory was enhanced when exercise occurred during memory retrieval. That is, there was no main effect for Encoding, *F*(1, 47) = 1.30, *p* = .26, η^2^ = .008, and no interaction between these factors,* F*(1, 47) = .01, *p* = .91, η^2^ < .001. However, there was a main effect for Retrieval, *F*(1, 47) = 5.56, *p* = .02, η^2^ = .03. Regarding the main effect for Retrieval, the number of words correctly recalled was higher when memory retrieval occurred during exercise compared to recall during rest, M_diff_ = .55 (95% CI: .08 – 1.02),* t* = 2.36, d = .34, *p* = .02.^5^ That is, and as shown in Fig. 2, RE and EE conditions resulted in similar levels of correct word recall that were higher than those in conditions ER and RR.

### Memory performance – incorrectly recalled words

**Fig. 3:**
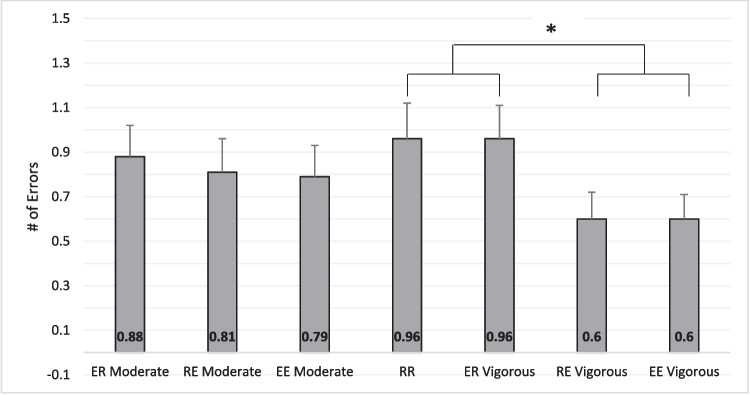
Number of errors (incorrectly recalled words) across the conditions. RR = rest-rest; RE = rest-exercise; ER = exercise-rest; EE = exercise-exercise. Moderate represents the moderate-intensity condition and Vigorous represents the vigorous-intensity condition. Error bars are standard errors of the mean. In a paired t-test, the mean number of errors was lower for the collapsed RE vigorous and EE vigorous conditions when compared to the collapsed RR and ER vigorous conditions

We confirmed that an overall increase in reported recall of words was the source of improved word recall when exercise occurred during retrieval as recall for incorrect words did not increase in the same manner correct recall did (see Fig. 3). There were no main or interaction effects for the 2 × 2 rmANOVAs for moderate intensity, all *p*s > .28. For vigorous-intensity, there was no main effect for Encoding, *F*(1, 47) < .001, *p* = 1.0, η^2^ < .001, or an interaction between Encoding and Retrieval, *F*(1, 47) < .001, *p* = 1.0, η^2^ < .001. In fact, the number of words incorrectly recalled was higher when memory retrieval occurred during rest when compared to during exercise, *F*(1, 47) = 9.13, *p* = .004, η^2^ = .06, M_diff_ = .35 (95% CI: .12 – .59),* t* = 3.0, d = .44, *p* = .004.

## Discussion

Our results demonstrate that memory performance was enhanced when retrieval occurred during exercise, but only when exercise was of vigorous intensity as compared to moderate intensity exercise. These results are in opposition to our original hypotheses and occurred regardless of encoding condition.

In contrast to previous research (often using cycling protocols with longer durations) suggesting that vigorous-intensity exercise reduces memory performance when the bout of exercise occurs during encoding (Jung et al., 2022, 2024; Loprinzi et al., 2019b; Roig et al., 2016; Tomporowski & Qazi, 2020), word recall was not reduced when encoding occurred during treadmill exercise regardless of intensity or recall conditions. Per the transient hypofrontality theory (Dietrich 2003, 2006), during vigorous-intensity exercise, attentional and metabolic resources may be reduced in prefrontal regions and allocated to sensory and motor cortices, increasing neural activation to sustain physical movement. More specifically, cycling at a self-perceived high intensity activates the posterior cingulate gyrus and precuneus (Fontes et al., 2015), both of which appear to play a role in modulating other motor areas, such as the primary motor cortex and supplementary motor area (Wenderoth et al., 2005). On the other hand, neural networks biased towards cognitive processes may be temporarily deactivated, reducing processing capacity for supported cognitive functions like working memory and consolidation (Jung et al., 2023). For example, Fontes et al. (2020) showed a reduction in activity of the dorsolateral prefrontal cortex, middle temporal gyrus, and parahippocampal gyrus during high-intensity cycling, and similarly, Kemppainen et al. (2005) showed that brain glucose levels decreased in the superior and middle frontal cortex during high-intensity cycling.

While studies support the hypofrontality hypothesis suggesting cognitive function can be reduced during vigorous-intensity exercise (Dietrich & Audiffren, 2011), it is possible a certain level of exercise must be reached before metabolic resources shift from memory-biased to motor support networks. Unless available resources exceed the amount necessary to simultaneously perform short-duration vigorous exercise and cognitive processing-based tasks, these resources may be more evenly distributed across different brain regions (e.g., motor cortex, prefrontal cortex, and hippocampus). Thus, this distribution may either enhance memory performance or, at the very least, not impair it (Audiffren, 2016).

The present experiment demonstrated that short-duration vigorous exercise during retrieval improved memory performance. Given the paucity of research evaluating potential mechanisms through which acute exercise may influence memory via retrieval-based processes (Loprinzi, Roig et al., 2021b), it is unclear as to why participants – in the present study – showed greater memory performance when they exercised at a vigorous-intensity during retrieval as compared to rest. As has been speculated elsewhere (Loprinzi et al., 2017; Loprinzi, Day et al., 2021a, Loprinzi, Roig et al., 2021b), higher exercise intensity may help facilitate the item-invariant (searching memory) aspects of memory retrieval. That is, exercise may facilitate the allocation of mental resources (perhaps via exercise-induced arousal/attention) that may be required for executive control-related monitoring processes to retrieve the encoded information successfully. Further, prior work demonstrates that during high-intensity exercise, the medial frontal gyrus becomes activated (Fontes et al., 2020), which is a key brain region in memory performance (Euston et al., 2012). The present study’s finding that short-duration (< 5 min), vigorous-intensity acute exercise performed during retrieval, coupled with recent research (Loprinzi, Day et al., 2021a, Loprinzi, Roig et al., 2021b) demonstrating that 20 min of vigorous-intensity acute exercise shortly before retrieval can improve memory, highlight that vigorous-intensity exercise may improve retrieval-based processes to enhance memory. This is a novel finding worthy of future exploration. The mechanisms underlying this effect, however, need to be carefully considered if indeed this is a replicable phenomenon. If exercise during retrieval activates prefrontal regions to facilitate item-invariant retrieval, then this mechanism would oppose the mechanism thought to impair encoding during vigorous-intensity exercise. As discussed above, when engaging in vigorous-intensity exercise during encoding, prefrontal cortex-dependent cognition can be impaired, which is thought to occur via a redistribution of metabolic and cognitive resources away from areas within the prefrontal cortex (Jung et al., 2022). But, hypofrontality induced deficits in cognition related to acute exercise are not universal and vary by exercise, cognitive task, and exercise duration and intensity (Cantelon & Giles, 2021) and some studies support widespread increased overall brain activity during exercise. Any effects of hypofrontality during exercise appear to be reversed post-exercise, with increased activation of several prefrontal cortical regions, improved mood, and increased executive function occurring post-running (Damrongthai et al., 2021). The interpretation of exercise-increased brain activation measured by glucose utilization (PET scans) is tempered by studies using cerebral flow, oxygenation, cortical activity, and fMRI (Perrey, 2013), which show hyperfrontality rather than hypofrontality, and by studies showing metabolic transitions from brain glucose to lactate utilization in the frontal cortex and other areas during exercise (Kemppainen et al., 2005). Any conflict between resource demands for cognition and exercise would be dependent on overlap between brain regions required for encoding, consolidation, and retrieval specific to the memory task used and the specific demands of the exercise task (Cabeza & Nyberg, 2000). The prefrontal cortex is not a homogenous region and different types of exercise and different memory tasks engage different parts of the prefrontal cortex and other regions (Cabeza & Nyberg, 2000), in particular, locomoting on a treadmill does not require brain input (Courtine et al., 2009). As alluded to above, even if similar brain resources are needed for exercise and a memory task, only when resources exceed the amount needed to perform both exercise and the cognitive task would there be interference. In our experiment, encoding might require more cognitive resources than retrieval (Anderson et al., 1998), and as such, there is greater competition for resources with exercise and encoding than there is for when exercise occurs during retrieval.

Another interesting observation of this experiment was that we did not observe a state-dependent learning effect. Consistent with the results of previous studies as well as per the tenets of state-dependent learning, it is conceivable that memory would be enhanced when state-related factors match between learning and retrieval, while memory would be reduced in a mismatched state between learning and retrieval (Miles & Hardman, 1998; Salas et al., 2011; Yanes et al., 2019; see Table 3 for further details on methodological differences between the present study and these prior studies including exercise protocols on state-dependent learning). One possible explanation of our observed null effect is that the retention interval, or the period between encoding and retrieval was too close, which may have enabled participants to better reinstate their state and preserve the encoded information regardless of whether state-related factors are incongruent.

**Table 3:** Methodological differences between the present study and prior studies on state-dependent learning in the exercise domain

References	Exercise parameters	Retention period	Task	Design and characteristics of participants	Results
Miles and Hardman (1998)	4 min high-intensity cycling on a bicycle ergometer	Approximately 5 min	36 word-list memory recall	Within-subject; 24 young adults (18–22 years)	A significant state-dependent effect
Salas et al. (2011)	10 min walk around the building at a brisk pace	Approximately 5 min	Free recall, judgement-of-learning magnitude, metamemory accuracy	Between-subject; 80 (20 per group) young adults (18–32 years)	No evidence of state-dependent effect, but memory recall was greater for W-W than S-S, S-W, and W-S.
Yanes et al. (2019)	15 min moderate-intensity walk on a treadmill	Approximately 20 min	15 word-list retrospective memory recall	Within-subject; 24 young adults (M_age_ = 21 years)	A significant state-dependent effect
Present Study	3 min moderate- and high-intensities exercise on a treadmill	Approximately 5.5 min	15 word-list retrospective memory recall	Within-subject; 48 young adults (18–25 years)	No evidence of state-dependent effect

W-W = walking-walking; S-S = sitting-sitting; S-W = sitting-walking; W-S = walking-sitting

One of the key challenges in state-dependent research is that just remembering details from the context, regardless of the physiological state, can be enough to reinstate the state and preserve memory (Smith & Vela, 2001). Because all visits in this study were in the same lab and same environmental context (i.e., encode and retrieve a list of words on a treadmill in the lab), coupled with a relatively short retention interval, it is possible that participants reinstated their encoding mental state during retrieval. This, in turn, may have attenuated or removed the potential state-dependent effect. Future work may wish to create a more drastic change in state or environmental context in the mismatch condition by, for example, altering the retention interval, having retrieval not occur on the treadmill but in a different environmental context, changing the physical posture (e.g., standing while exercising during encoding and then sitting during retrieval), and even possibly trying to alter the emotional or cognitive state between encoding and retrieval.

Additionally, the state condition controlled herein only includes the physiological state (i.e., heart rate). Mead and Ball (2007) demonstrated greater memory performance when musically induced mood was congruent during encoding and retrieval. Considering this finding, it would be intriguing for future studies to include assessments of both affective (e.g., mood, arousal) and physiological states during encoding and retrieval. For example, participants could self-report their mood and arousal using the Feeling Scale and Felt Arousal Scale (Rejeski et al., 1987; Svebak & Murgatroyd, 1985), asking how they emotionally feel and how aroused they are at the moment during specific activities. Such alterations in mood and arousal are regarded as key components affecting the strength and stability of memory processes (Bower et al., 1978). Plus, assessment of these parameters may allow for another interesting idea to be explored. Acute moderate-intensity exercise generally induces a more positive affective state when compared to vigorous-intensity exercise (Ekkekakis et al., 2008). Although speculative, perhaps processes involved in memory retrieval during short-duration, vigorous-intensity exercise could favorably alter how one cognitively appraises and interprets exercise-induced interoceptive changes (e.g., increased ventilation). This temporary distraction away from the typically observed negative affective responses (of vigorous exercise) may allow for an adequate allocation of resources for item invariant retrieval. On the other hand, moderate-intensity exercise often induces a positive affective state that the individual might wish to maintain. In this context, trying to maintain both the desired mental state and favorable interoceptive and psychological responses to moderate-intensity exercise could intensify the challenge of accessing memory for successful retrieval. This phenomenon is likely more pronounced in such a dual-task scenario. Of course, the alternative is equally likely, in that the positive affective and arousal states resulting from moderate-intensity exercise could improve cognitive processing by directing attentional resources more towards the cognitive task. Future work may wish to explore these exciting possibilities.

One limitation of our study is that the duration of exercise immediately before memory encoding and retrieval were not identical – participants exercised for 2 min before commencing encoding and exercised for 3 min before commencing retrieval. We anticipated that encoding would take approximately one minute, equaling about 3 total minutes of exercise that included exercise before and during encoding. This was our reasoning for having participants exercise for 3 min before retrieval occurred. Relatedly, in the present experiment, exercise and encoding/retrieval did not commence exactly at the same time. The reason for this was because encoding and retrieval did not last up to 5 min in duration (the duration of exercise). If future work demonstrates that even shorter durations of acute exercise benefit memory, then such work may wish to have the exercise bout and duration match the exact timescale of encoding and retrieval. The field would also benefit by evaluating whether memory retrieval while exercising at vigorous intensity varies as a function of the duration of exercise. It is possible that the beneficial effects of acute exercise during retrieval may reverse with longer durations of exercise. Although not specific to memory (combined results from a variety of cognitive tasks), a previous meta-analysis (Chang et al., 2012) evaluated the timing of the cognitive task during an exercise bout (within the first 10 min, 11–20 min, and beyond 20 min of exercise) on overall cognitive performance. The authors reported a moderating effect; the influence of the timing of the cognitive task during exercise being negligible, detrimental, and beneficial across the aforementioned time windows, respectively. Future work could extend the duration of exercise to identify at what specific time points memory is impaired based on when encoding and retrieval occurs during the bout of exercise.

Notable strengths of this experiment are multifold. First, our study included a relatively large within-subjects sample of 48 participants (twice the sample size of prior studies; Miles & Hardman, 1998; Salas et al., 2011; Yanes et al., 2019), with the utilization of a maximal bout of exercise in the first visit to determine the heart rate thresholds used for setting the individualized exercise intensities and including multiple exercise intensities for the subsequent exercise sessions. Second, our study examined both moderate- and vigorous-intensity exercise during both memory encoding and retrieval, which is infrequently evaluated in exercise-memory experiments. Similarly, given that most of the work on this topic has focused on the effects of much longer exercise durations on memory, our findings support evidence that short-duration, vigorous-intensity acute exercise during memory retrieval can contribute to memory enhancement. Future work would benefit by replicating the current findings while identifying potential causal mechanisms of retrieval-based processes that may be augmented with exercise. As portable brain imaging continues to improve, the bias towards cycling studies (Kemppainen et al., 2005) may be reduced. In such replication attempts, future work may wish to consider employing a fully crossed design. What is missing from the current design is encoding during vigorous-intensity exercise and retrieval during moderate-intensity exercise and vice versa. Evaluating a fully crossed design such as this will allow one to more thoroughly determine whether subtle or drastic differences in the mismatching conditions are necessary to provide support for state-dependency theory in the exercise domain.

### Practical applications of the present experiment

The present study demonstrates that a short bout of exercise during recall may improve memory performance. Although not all research has to have immediate practical applications, the findings of the present experiment may have implications on the potential time-saving effects of very short-duration exercise on free-recall memory performance. This finding also has implications for the educational domain, as incorporating movement during learning/retrieval may improve classroom behavior and memory in young populations (Lucht & Heidig, 2013). Further, with the advent of treadmills with a desk workstation and cycling desks, students will have more opportunities to engage in movement during studying and retrieval practice, which may help to facilitate long-term memory performance.

Additionally, even after learning information, a short, intense warm-up or bout of exercise, while thinking back to the learned information, may help facilitate retrieval processes and reconsolidation of the memory. In alignment with the frameworks of embodied memory (Iani, 2019) and state-dependent memory (Tulving & Thomson, 1973), the act of exercise may not even be necessary; retrieving this previously learned information while thinking about exercise may facilitate further recall of the learned information.

## Conclusion

In conclusion, our findings suggest that a short-duration (< 5 min) bout of vigorous-intensity exercise during memory retrieval can improve memory performance. State-dependent learning was not needed to observe these beneficial effects of vigorous-intensity exercise on memory.

## Data Availability

Data and materials are available upon request.

## Footnotes

1 An interaction between these two factors would be evidence of state-dependency.

2 Moderate-to-vigorous physical activity (MVPA) was assessed from the Physical Activity Vitals Sign (PAVS) questionnaire, asking participants to report their daily MVPA behavior.

3 With utilizing a within-subject design, it is possible that, due to the repeated visits, participants may have anticipated the condition or became used to the dual task of exercising while encoding/retrieving. To determine if this possibility attenuated state-dependent learning, we treated condition as a between-subject factor, only analyzing data from the participant’s first visit. With correct recall as the outcome, and with condition (all seven) as a between-subject factor, no differences were observed, *F*(6, 41) = .40, *p* = .87. Similarly, with condition as a two-level between subject factor (matched context (RR, EE Moderate, EE Vigorous) versus mismatched context (ER Moderate, RE Moderate, ER Vigorous, RE Vigorous), no differences were observed, *F*(1, 46) = .003, *p* = .96. Thus, these results suggest that any potential anticipation of the condition/visit may have had minimal effects on influencing state-dependent learning.

4 We also considered the possibility of whether the null state-dependent learning could be a result of the participant’s habitual physical activity (MVPA) or a proxy measure of their cardiorespiratory fitness (duration lasted on the treadmill for the initial max visit). However, we did not observe any statistically significant Encoding × Retrieval × MVPA, or Encoding × Retrieval × fitness interactions, *p*s > .05.

5 We computed an additional analysis that included both moderate- and vigorous-intensity exercise in the model, with an omission of the RR condition. This analysis consisted of a 2 (Exercise: Moderate v Vigorous) × 3 (State: RE, ER, EE) ANOVA. This analysis demonstrated no main effect for Exercise, *F*(1, 47) = .01, *p* = .91, and no interaction between Exercise and State, *F*(2, 94) = .72, *p* = .49, but suggestive evidence of a main effect for State, *F*(2, 94) = 3.09, *p* = .05. Post-hoc analyses demonstrated that correct recall was higher for RE than ER, M_diff_ = .54, *p* = .03, and that correct recall was lower for ER than EE, M_diff_ = -.56, *p* = .04. However, there was no statistically significant difference between RE an EE, M_diff_ = -.02, *p* = .93.
